# Management of secondary hyperparathyroidism: practice patterns and outcomes of cinacalcet treatment with or without active vitamin D in Austria and Switzerland – the observational TRANSIT Study

**DOI:** 10.1007/s00508-016-1153-z

**Published:** 2017-01-13

**Authors:** Wolfgang Pronai, Alexander R. Rosenkranz, Andreas Bock, Renate Klauser-Braun, Christine Jäger, Gunther Pendl, Margit Hemetsberger, Karl Lhotta

**Affiliations:** 1 Department of Internal Medicine, Dialysis Unit, Hospital of the Brothers of Saint John of God, Johannes von Gott Platz 1, 7001 Eisenstadt, Austria; 2 Department of Internal Medicine, Clinical Division of Nephrology, Medical University of Graz, Graz, Austria; 3 Abteilung Nephrologie, Kantonsspital Aarau, Aarau, Switzerland; 4 Sozialmedizinisches Zentrum Ost – Donauspital, Vienna, Austria; 5 Amgen GmbH, Vienna, Austria; 6 Amgen AG, Zug, Switzerland; 7 hemetsberger medical services, Vienna, Austria; 8 Department of Nephrology and Dialysis, Academic Teaching Hospital Feldkirch, Feldkirch, Austria

**Keywords:** Cinacalcet, Secondary hyperparathyroidism, Cinacalcet, Secondary hyperparathyroidism, Treatment pattern, Clinical practice, Observational study

## Abstract

Secondary hyperparathyroidism is a complex disorder requiring an individualized multicomponent treatment approach. This study was conducted to identify treatment combinations used in clinical practice in Austria and Switzerland and the potential to control this disorder. A total of 333 adult hemodialysis and peritoneal dialysis patients were analyzed. All patients received conventional care prior to initiation of a cinacalcet-based regimen. During the study, treatment components, e.g. cinacalcet, active vitamin D analogues and phosphate binders, were adapted to individual patient requirements and treatment dynamics were documented. Overall, the mean intact parathyroid hormone (iPTH) increased from 64.2 pmol/l to 79.6 pmol/l under conventional therapy and decreased after cinacalcet initiation to 44.0 pmol/l after 12 months (mean decrease between baseline and 12 months −45%). Calcium remained within the normal range throughout the study and phosphorus ranged around the upper limit of normal. The Kidney Disease: Improving Global Outcomes (KDIGO) target achievement for iPTH increased from 44.5% of patients at baseline to 65.7% at 12 months, corrected calcium from 58.9% to 51.9% and phosphorus from 18.4% to 24.4%. On average, approximately 30% of patients adapted their regimen from one observation period to the next. The reasons for changing a given regimen were to attain or maintain any of the bone mineral markers within recommended targets and to avoid developments to extreme values. Some regional differences in practice patterns were identified. No new safety signals emerged. In conclusion, cinacalcet appears to be a necessary treatment component to achieve recommended targets. The detailed composition of the treatment mix should be adapted to patient requirements and reassessed on a regular basis.

**Electronic supplementary material:**

The online version of this article (doi: 10.1007/s00508-016-1153-z) contains supplementary material, which is available to authorized users.

## Introduction

Secondary hyperparathyroidism (SHPT) is a severe and progressive disorder frequently observed in patients from an early stage of chronic kidney disease (CKD) onwards. At the time this study was planned and initiated, in the years 2009/2010, the principles of therapy of SHPT were profoundly questioned and changed. A new theory of the pathogenesis of SHPT placed more emphasis on the control of serum phosphorus levels [1]. This newer theory views the CKD-induced impaired activation of vitamin D not as the cause of SHPT but as an adaptive reaction to processes occurring much earlier in the cascade of events. According to this theory phosphorus retention in the failing kidney leads to increases in circulating fibroblast growth factor 23 (FGF-23) levels. Together with the Klotho protein FGF-23 tries to restore effective renal phosphorus clearance. In addition, FGF-23 blocks the production of active vitamin D. Only in the very late stages of CKD, when no sufficient renal function remains and phosphorus clearance can no longer be supported by intrinsic mechanisms, the PTH-calcium-vitamin D axis as described in the “trade-off” comes into play [2]. Following this reasoning, SHPT treatment should primarily be based on phosphorus restriction in combination with physiologic doses of active vitamin D analogues. In patients where phosphate binders and physiologic vitamin D doses alone are insufficient to control parathyroid hormone (PTH), calcimimetics should be considered as first-line therapy to control serum PTH [1]. On the other hand, the Kidney Disease: Improving Global Outcomes (KDIGO) Chronic Kidney Disease – Mineral and Bone Disorder (CKD-MBD) guidelines issued in 2009 [3] were less stringent with respect to PTH target levels than the previously used National Kidney Foundation Kidney Disease Outcomes Quality Initiative (NKF-KDOQI™) guidelines [4]. The 2003 NKF-KDOQI™ clinical practice guidelines for bone mineral metabolism and disease in CKD defined stringent target ranges for the key parameters of bone mineral metabolism (iPTH: 16.5–33.0 pmol/l; serum phosphorus: 1.1–1.78 mmol/l; serum calcium: 2.1–2.37 mmol/l; corrected calcium-phosphorus product <4.4 mmol²/l²) [4]. In 2009, the KDIGO clinical practice guidelines for the diagnosis, evaluation, prevention and treatment of CKD-MBD changed their focus towards defining ranges of extreme risk that should be avoided [3]. For intact PTH (iPTH), the recommended safe range was defined as 2–9 times the upper limit of normal (ULN) of the assay used; calcium is recommended to be maintained in the normal range and elevated phosphorus should be lowered toward the normal range. These recommendations allow some individualization of treatment much welcomed by the medical community.

At the time this study was planned a survey evaluating the quality of CKD-MBD treatment in Austria (Austrian Dialysis and Transplant Registry; “QUASI” 2008, www.nephro.at), revealed that approximately 30% of Austrian patients received cinacalcet as a monotherapy, without concomitant administration of active vitamin D compounds. From the survey, however, it was not clear, whether cinacalcet was used as a monotherapy already at cinacalcet initiation or if active vitamin D was initially co-administered and discontinued later. One aim of this study therefore was to determine the proportion of patients where cinacalcet was initiated as a monotherapy or in combination with active vitamin D. Another aim of this study was to identify treatment combinations used in clinical practice and their potential to control CKD-MBD.

## Methods

### Study design

The **TR**eatment pr**A**ctice for ma**N**agement of **S**HPT with c**I**nacalcet and vi**T**amin D (TRANSIT) study was a single-arm, partly retrospective, partly prospective observational study conducted in Austria and Switzerland. The observation period was 18 months: data were collected 6 months prior to cinacalcet initiation (month -6) or from start of dialysis onwards in patients with less than 6 months of dialysis vintage until 12 months after cinacalcet initiation (month 12). There was no control group. The study design allowed retrospective, intraindividual, longitudinal control. This study was non-interventional, i. e. no study-related changes to routine clinical treatment, changes in therapy or co-medication, diagnostic work-up or monitoring of participating patients were foreseen in the study protocol, nor were additional hospital visits required for the sole purpose of meeting study requirements. Patients were only included if cinacalcet treatment was initiated prior to study inclusion to avoid initiation of the drug for the purpose of participation in this study. Cinacalcet treatment was conducted according to the approved indications and the judgment of the treating physician.

### Eligibility

Hemodialysis and peritoneal dialysis patients ≥18 years of age and indicated for treatment with cinacalcet according to the label were included, if they had been initiated on cinacalcet for no longer than 3 months prior to inclusion in this study. Patients contraindicated for treatment with cinacalcet as per label, patients participating in a clinical trial expected to confound the endpoints of this study or patients with planned kidney transplantation within the observation period of this study were excluded.

### Participating centers and sample size estimation

The total planned patient number was approximately 330, based on experience from previous observational studies in the participating countries. The similarly designed Austrian Evaluation of the Clinical Use of Mimpara® in Hemodialysis and Peritoneal Dialysis Patients, an Observational Study (ECHO) enrolled 320 patients [5]. Dialysis centers were selected on the basis of relevant experience and an estimated recruitment capacity of a minimum of five patients per center. Representativeness of the totality of selected centers was defined by criteria, such as geographical region or type of center (e.g. urban, rural, academic and non-academic).

### Data collection

Data of eligible patients were collected from their charts. Centers were asked to include all patients newly initiated on cinacalcet within 3 months prior to study start until the end of the enrollment period or until the maximum number of 20 patients allowed per center was reached. Eligible patients were included in the study sequentially by date of cinacalcet initiation. Patients were informed about the collection of the data for the purpose of this study and were required to provide written informed consent. For Austria, ethics committee approval was obtained centrally from the institutional review board of the Medical University of Graz, Austria. For Switzerland no ethics committee approval was legally required at the time of study conduct.

For the purpose of documentation, electronic or paper case report forms (CRF) were completed by the treating physician. Internal integrity and logic of data were assured by checking the returned CRF for completeness, plausibility and obvious discrepancies. An additional quality check encompassing 30% of data in 10% of patients was conducted on site under strict maintenance of data privacy by the means of indirect methods (interview) to ensure consistency with source documents. Only the treating physician had direct access to patient files.

### Study parameters

The primary study parameter was the proportion (%) of end-stage renal disease patients with CKD-MBD, who were initiated on a regimen of cinacalcet monotherapy without concomitant administration of active vitamin D compounds. Secondary study parameters were mineral metabolite trajectories, NKF-KDOQI™ and KDIGO target achievement, usage patterns of cinacalcet and relevant concomitant medications (e.g. active vitamin D compounds and phosphate binders), patient characteristics and demographics and adverse event (AE) reports.

Since the KDIGO guidelines do not provide exact target ranges, we used the ranges provided by the Austrian Dialysis and Transplant Registry, who have standardized normal ranges over all assays used for the parameters of interest in Austria and set recommended target ranges for iPTH (12.72–63.6 pmol/l), phosphorus (1.13–1.48 mmol/l) and calcium (corrected 2.1–2.4 mmol/l) [3, 6]. For NKF-KDOQI™ the published target ranges were used (iPTH 16.5–33.0 pmol/l, phosphorus 1.13–1.78 mmol/l, corrected calcium 2.1–2.37 mmol/l, and corrected calcium-phosphorus product <4.44 mmol²/l²) [4].

### Treatment assignment

Classification of patients with respect to treatment regimen type was based on their medication use at each timepoint of interest. These regimen types comprised cinacalcet monotherapy, cinacalcet plus low dose vitamin D (defined as a maximum dose of 2 μg paricalcitol = 1 μg doxercalciferol = 1 μg alfacalcidol = 0.5 μg calcitriol administered intravenously with each dialysis session, i. e. three times weekly, or a daily oral dose of 1 μg paricalcitol = 0.5 μg alfacalcidol = 0.25 μg calcitriol), cinacalcet plus high dose vitamin D (doses higher than the ones defined as low dose vitamin D), vitamin D monotherapy or no SHPT therapy. All patients in all groups were allowed to receive concomitant phosphate binders. Patient flow charts were prepared to illustrate the dynamics of CKD-MBD treatment over time.

### Statistical analysis

Patients were analyzed overall and by different treatment regimen types. No formal hypothesis was tested. The full analysis set (FAS) comprised all enrolled patients who were initiated on cinacalcet and who received at least one dose of cinacalcet. Categorical variables are summarized as the percentage of patients in each category. Continuous variables are presented as means, standard deviations (SD), medians, minimum and maximum values and 95% confidence intervals (CI) of means for the overall group are presented. Only available entries were analysed and no last observation carried forward (LOCF) was conducted. In the electronic case report form (eCRF) the following conversion factors for conventional to SI units were programmed: albumin g/d × 10 = g/l, Ca (total, ionized) mg/dl × 0.25 = mmol/l, Ca × *P* mg^2^/dl^2^ × 0.08 = mmol^2^/l^2^, iPTH pg/ml × 0.1053 = pmol/l and *P* mg/dl × 0.323 = mmol/l.

For statistical analysis SPSS software, V.17 (IBM, Armonk, NY) was used.

## Results

### Study population

Between February 2010 and December 2013 data from a total of 335 patients were collected. A total of 333 patients (Austria *n* = 165; Switzerland *n* = 168) were analyzed and 2 patients were excluded from the analysis: 1 patient did not receive cinacalcet and 1 patient was <18 years of age. At month 12, data were available from 241 patients (73.4%). Patient status at the end of documentation was available from 333 patients: 238 (71.5%) had full documentation of 12 months of observation period and completed end of documentation status. Of the patients 66 (19.8%) had incomplete documentation and provided a reason for discontinuation of which 27 (8.1%) died, 18 (5.4%) received a transplant, 6 (1.8%) moved, 15 (4.5%) had other reasons and 29 patients (8.7%) did not provide any status.

Patient demographics are shown in Table 1. Overall, almost all patients received hemodialysis (96.1%), 61.4% of patients were male and 93.1% were of Caucasian origin. The mean (SD) age was 60.8 (14.4) years and the two most prevalent etiologies of CKD were diabetes mellitus (29.4%) and vascular nephropathy (28.8%).

**Table 1:** Patient demographics and characteristics

Parameter	Overall	Austria	Switzerland
Gender, *n* (%)			
*N*	332	165	167
Male	204 (61.4)	103 (62.4)	101 (60.5)
Female	128 (38.6)	62 (37.6)	66 (39.5)
Ethnicity, *n* (%)			
*N*	333	165	168
Caucasian	310 (93.1)	161 (97.6)	149 (88.7)
Black/African American	13 (3.9)	1 (0.6)	12 (7.1)
Asian	8 (2.4)	3 (1.8)	5 (3.0)
Hispanic/Latino	2 (0.6)	0	2 (1.2)
Age, years			
*N*	333	165	168
Mean (SD)	60.8 (14.4)	59.4 (14.9)	62.1 (13.7)
Median (min, max)	62.0 (22, 89)	61.0 (22, 88)	64.0 (23, 89)
Weight, kg			
*N*	331	165	166
Mean (SD)	78.3 (17.6)	81.3 (16.9)	75.4 (17.8)
Median (min, max)	77.6 (36, 142)	80.0 (47, 139)	74.0 (36, 142)
Height, cm			
*N*	321	165	156
Mean (SD)	169.2 (9.2)	169.9 (9.1)	168.5 (9.3)
Median (min, max)	170.0 (139, 198)	170.0 (130, 198)	169.0 (130, 189)
Primary etiology of CKD, *n* (%)			
*N*	333	165	168
Diabetes mellitus	98 (29.4.5)	57 (34.5)	41 (24.4)
Vascular nephropathy	96 (28.8)	44 (26.7)	52 (31.0)
Glomerulonephritis	38 (11.4)	16 (9.7)	22 (13.1)
Polycystic nephropathy	33 (9.9)	10 (6.1)	23 (13.7)
Interstitial nephropathy	9 (2.7)	3 (1.8)	6 (3.6)
Other	83 (24.9)	48 (29.1)	35 (20.8)
Dialysis method, *n* (%)			
*N*	333	165	168
Hemodialysis	320 (96.1)	163 (98.8)	157 (93.5)
Peritoneal dialysis	13 (3.9)	2 (1.2)	11 (6.5)
iPTH at baseline, pmol/l			
*N*	301	149	152
Mean (SD)	79.6 (49.7)	76.1 (39.1)	83.0 (58.2)
Median (min, max)	68.4 (11.2, 438.0)	66.2 (22.1, 260.3)	69.9 (11.2, 438.0)
Calcium (corrected) at baseline, mmol/l			
*N*	241	116	125
Mean (SD)	2.27 (0.22)	2.21 (0.21)	2.32 (0.20)
Median (min, max)	2.26 (1.51, 2.82)	2.23 (1.53, 2.82)	2.29 (1.51, 2.81)
Phosphorous at baseline, mmol/l			
*N*	321	158	163
Mean (SD)	1.87 (0.43)	1.94 (0.44)	1.81 (0.42)
Median (min, max)	1.81 (1.00, 3.09)	1.88 (1.16, 3.09)	1.77 (1.00, 2.93)
PTH trigger to initiate cinacalcet, *n* (%)			
*N*	323	165	158
iPTH > 33 pmol/l	150 (46.4)	87 (52.7)	63 (39.9)
iPTH > 9x ULN	40 (12.4)	15 (9.1)	25 (15.8)
Increasing iPTH trend	102 (31.6)	59 (35.8)	43 (27.2)
Patient-/center-specific iPTH value	31 (9.6)	4 (2.4)	27 (17.1)

*CKD* chronic kidney disease, *N* number of patients with available data, *SD* standard deviation, *ULN* upper limit of normal of the assay used, *PTH* parathyroid hormone, *iPTH* intact parathyroid hormone

Percentages are based on the number of patients with valid entries

### CKD-MBD treatment patterns

**Fig. 1:**
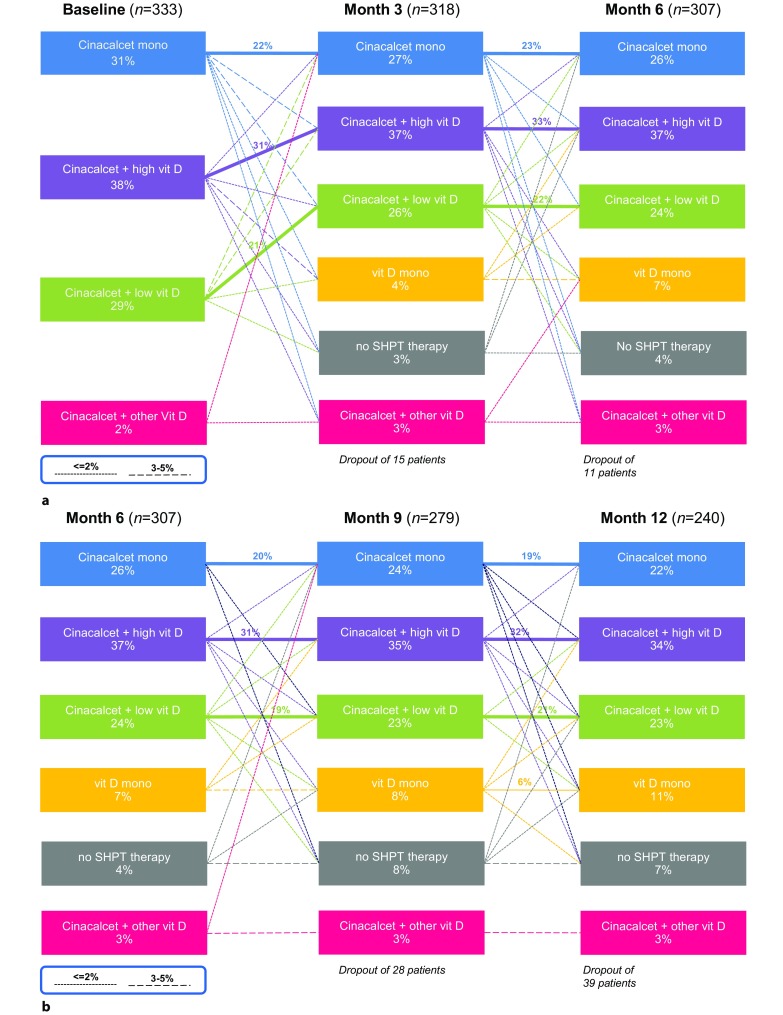
Group dynamics of SHPT therapies over time. **a** Baseline to month 6. **b** Month 6 to month 12. Cinacalcet mono, subgroup of patients receiving cinacalcet monotherapy at the specific point in time; cinacalcet + high vit D, subgroup of patients receiving cinacalcet plus high dose active vitamin D compounds at the specific point in time; cinacalcet + low vit D, subgroup of patients receiving cinacalcet plus low dose active vitamin D compounds at the specific point in time (low dose active vitamin D was defined as a maximum of 2 µg intravenous paricalcitol three times weekly or equivalent)

At cinacalcet initiation (i.e. baseline), 31.2% of patients (*n* = 104) started cinacalcet without concomitant vitamin D therapy (primary outcome measure). Table 1 lists the iPTH triggers to initiate cinacalcet at baseline. The two most important triggers were an iPTH > 33 pmol/l (46.4%, *n* = 150) and an increasing iPTH trend (31.6%, *n* = 102). The two most important primary reasons to start cinacalcet were to reduce PTH in patients with hyperphosphatemia (56.3%, *n* = 174) or to reduce PTH in patients with normophosphatemia and normocalcemia (21.7%, *n* = 67; Figure S2). In addition to cinacalcet monotherapy, 37.8% of patients (*n* = 126) received cinacalcet in combination with high dose vitamin D and 28.5% (*n* = 95) received cinacalcet plus low dose vitamin D. Over time, the proportion of patients assigned to these groups remained relatively stable, with approximately 20% of patients remaining on cinacalcet monotherapy, approximately 30% remained on cinacalcet plus high dose vitamin D, and approximately 20% remained on cinacalcet plus low dose vitamin D throughout the 12-month study duration. On an individual level, however, regimens were adjusted to meet patients’ needs (Fig. 1, Table S1). Starting with month 3, patients interrupting or permanently discontinuing cinacalcet emerge. The most important primary reasons to discontinue or interrupt cinacalcet were PTH suppression (39.4%, *n* = 39) and other reasons (37.4%, *n* = 37). Of the patients 12 (12.1%) stopped or interrupted cinacalcet because they had reached the target range for iPTH (Figure S2.B). At month 12, 198 (82.6%) out of 241 patients with available values received cinacalcet. Patients stopping cinacalcet continued on two possible regimens: vitamin D monotherapy or no SHPT therapy, both of which included optional phosphate binders.

The median daily cinacalcet dose was 30.0 mg at all timepoints overall and in all groups that had cinacalcet as their treatment backbone. The mean daily doses increased from 30.7 mg (95% CI ± 0.83 mg) overall at baseline to 45.4 mg (95% CI ± 3.37 mg) at month 12 (Table S2). The highest mean dose at month 12 was observed in the cinacalcet monotherapy group with 50.9 mg/week (baseline: 30.4 mg/week), the lowest in the cinacalcet plus low dose vitamin D group with 39.8 mg/day (baseline: 30.0 mg/day). In patients receiving active vitamin D analogues the majority of patients received oral calcitriol (ranging over time between 60% and 65% of those patients with valid vitamin D doses), followed by i. v. alfacalcidol (11–15%), i. v. paricalcitol (9–13%), and oral alfacalcidol (5–9%). The median weekly vitamin D dose was 6.0 µg i. v. paricalcitol equivalents at all timepoints overall and in the cinacalcet plus low dose vitamin D group, 14.0 µg in the cinacalcet plus high dose vitamin D group and between 12.0 and 14.0 µg in the vitamin D monotherapy group. The mean weekly doses ranged around 11 µg overall throughout the study, with approximately 5 µg in the cinacalcet plus low dose vitamin D group and 14 to 15 µg in the cinacalcet plus high dose vitamin D group.

All treatment groups optionally included phosphate binders (Table S2). Overall, the proportion of patients receiving phosphate binders remained stable between 60% and 66% of patients, with approximately 20% of patients receiving more than one phosphate binder. The proportion of patients receiving calcium-based phosphate binders varied over time between 47 and 51%; the proportion of patients receiving non-calcium-based phosphate binders had an increasing trend from 42% at month −3 to 53% at month 12. This category includes a relatively constant group of patients (12 to 16%) receiving aluminium-based phosphate binders (Table S2).

### Mineral markers over time

**Fig. 2:**
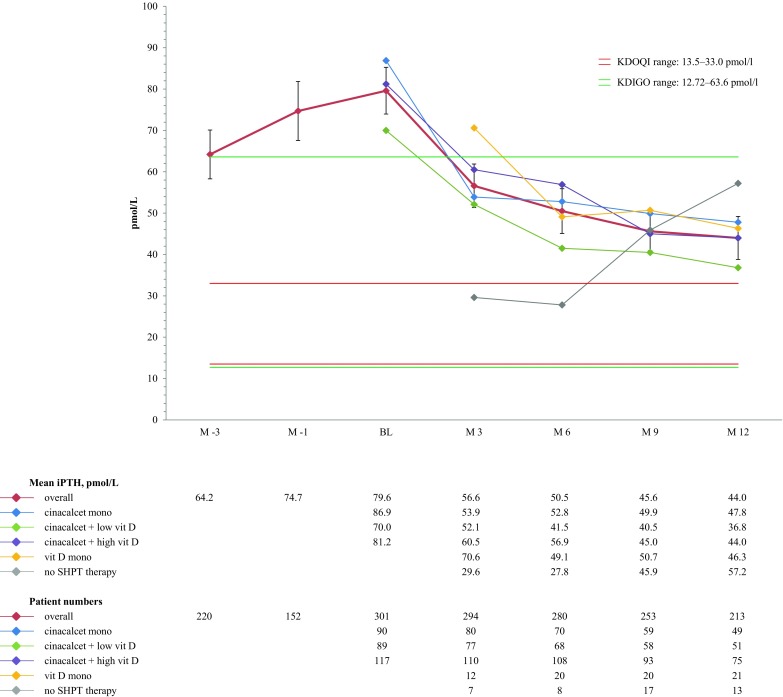
Bone mineral markers over time. Median iPTH over time (pmol/l)

Overall, mean iPTH increased from 64.2 pmol/l (95% CI ± 5.90) 3 months before baseline to 79.6 pmol/l (95% CI ± 5.64) at baseline and decreased thereafter to 44.0 pmol/l (95% CI ± 5.20) at month 12. Among the different study groups, the mean baseline iPTH value in patients with cinacalcet monotherapy or cinacalcet plus high dose vitamin D was higher than in patients with cinacalcet plus low dose vitamin D patients. At study end, iPTH values were within target in all groups (Fig. 2). Overall, the mean percentage decrease in iPTH between baseline and month 12 was −45%; the largest reduction was found in patients receiving cinacalcet monotherapy (−45%; Table 2). At month 3, the first patients interrupted cinacalcet therapy, receiving either vitamin D monotherapy or no SHPT therapy at all. In the vitamin D monotherapy group, mean iPTH decreased from 70.6 mmol/l (range 6.0 to 241.9) at month 3 to 46.3 mmol/L (range 6.6 to 220.6) at month 12. In the no SHPT therapy group, patients had very low PTH values at month 3 (mean 29.6 mmol/l; range 4.2 to 100.1), which rose to 57.2 mmol/l (range 1.6 to 209.5) at month 12 (Fig. 2).

**Table 2:** Mean percentage changes in iPTH

	Overall		Austria		Switzerland	
	Baseline to month 12 (%)	Month 6 to month 12 (%)	Baseline to month 12 (%)	Month 6 to month 12 (%)	Baseline to month 12 (%)	Month 6 to month 12 (%)
Overall	−45 (*n* = 301)	−13 (*n* = 280)	−46 (*n* = 149)	−9 (*n* = 146)	−43 (*n* = 152)	−16 (*n* = 134)
Cinacalcet mono	−45 (*n* = 90)	−9 (*n* = 70)	−44 (*n* = 35)	9 (*n* = 31)	−45 (*n* = 55)	−19 (*n* = 39)
Cinacalcet + low vit D	−47 (*n* = 89)	−11 (*n* = 68)	−53 (*n* = 39)	−21 (*n* = 35)	−42 (*n* = 50)	2 (*n* = 33)
Cinacalcet + high vit D	−46 (*n* = 117)	−23 (*n* = 108)	−42 (*n* = 74)	−14 (*n* = 60)	−52 (*n* = 43)	−31 (*n* = 48)
Vit D mono^a^	–	−6 (*n* = 21)	–	19 (*n* = 12)	–	−22 (*n* = 9)
No SHPT therapy^a^	–	106 (*n* = 13)	–	63 (*n* = 6)	–	90 (*n* = 7)

^a^Patients receiving vitamin D monotherapy or no SHPT therapy first appear at month 3

**Fig. 3:**
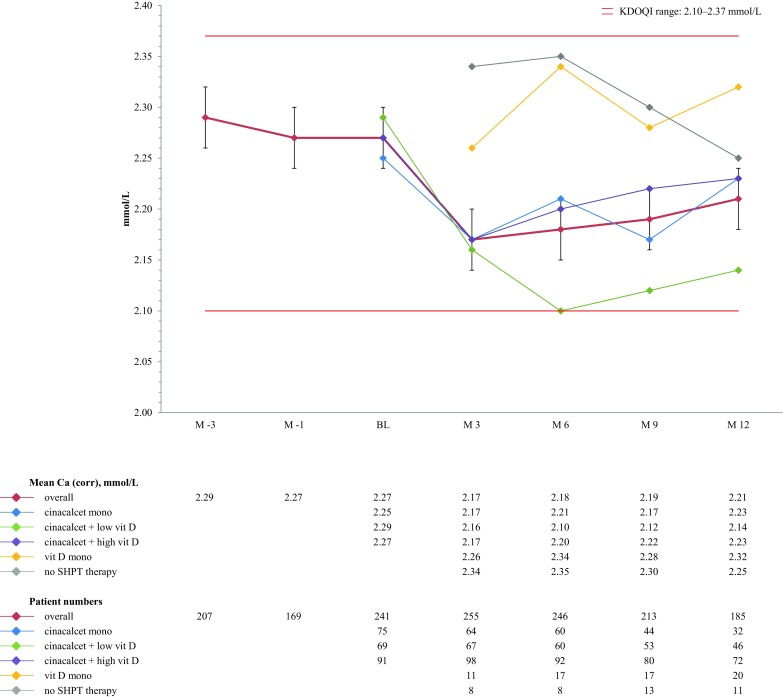
Median calcium (corrected) over time (mmol/l)

**Fig. 4:**
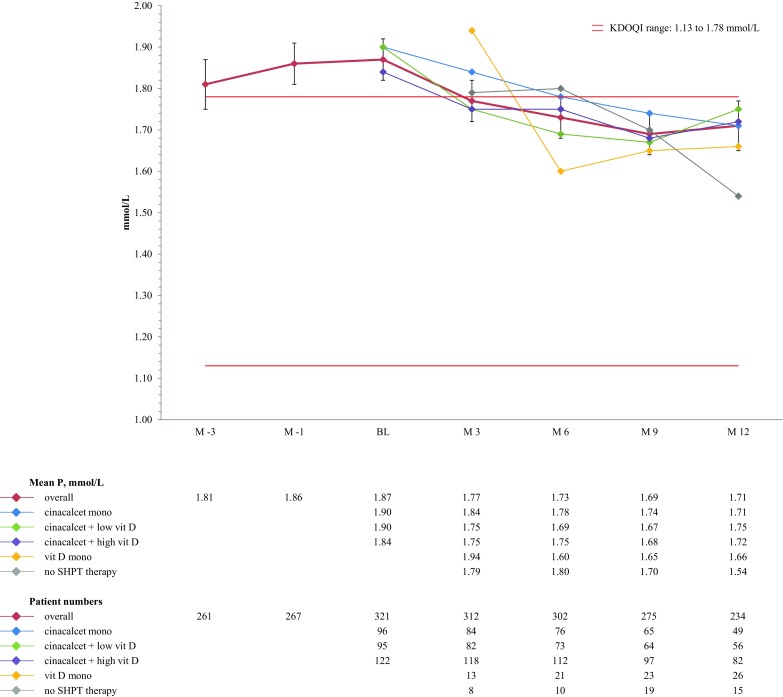
Median phosphorus over time (mmol/l)

Note: since the KDIGO guidelines do not provide exact target ranges, reference ranges provided by the Austrian Dialysis and Transplant Registry were used (12.72–63.6 pmol/l), phosphorus (1.13–1.48 mmol/l), and calcium (corrected; 2.1–2.4 mmol/l) [3, 6]. For NKF-KDOQI™ the published target ranges were used (iPTH: 16.5–33.0 pmol/l, phosphorus: 1.13–1.78 mmol/l, corrected calcium: 2.1–2.37 mmol/l, and corrected calcium-phosphorus product: <4.44 mmol²/l²) [4]. Cinacalcet mono, subgroup of patients receiving cinacalcet monotherapy at the specific point in time; cinacalcet + high vit D, subgroup of patients receiving cinacalcet plus high dose active vitamin D compounds at the specific point in time; cinacalcet + low vit D, subgroup of patients receiving cinacalcet plus low dose active vitamin D compounds at the specific point in time (low dose active vitamin D was defined as a maximum of 2 µg intravenous paricalcitol three times weekly or equivalent).

Mean corrected serum calcium remained within target over time with a trend towards higher calcium in patients receiving vitamin D monotherapy or no SHPT therapy compared to the other groups (Fig. 3). Mean serum phosphorus ranged around the upper limit of the recommended target range during the entire study period (Fig. 4).

### Target achievement

**Fig. 5:**
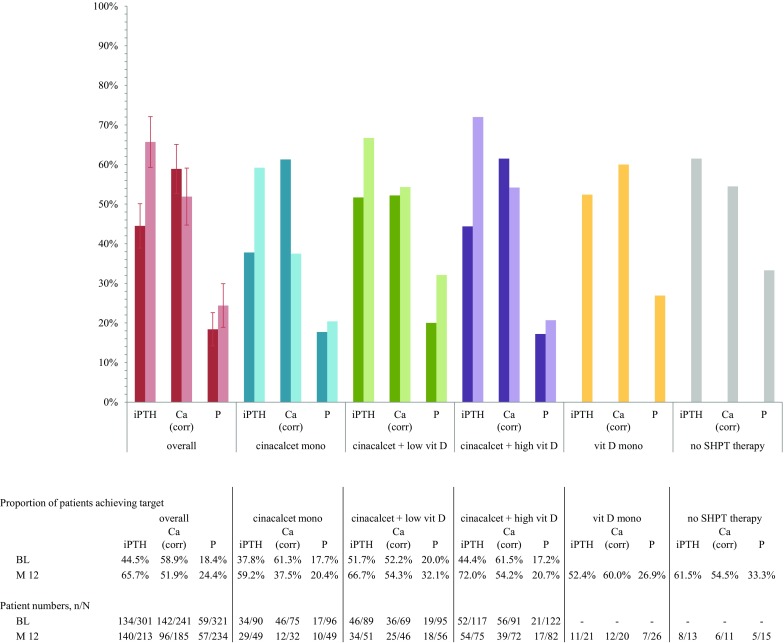
Target achievement at baseline and at month 12, overall and by subgroups. Proportion of patients (95% CI) reaching KDIGO recommended target ranges, based on “normal” values from the Austrian dialysis and transplantation registry [5]: iPTH (12.72–63.6 pmol/l), phosphorus (1.13–1.48 mmol/l), and calcium (corrected; 2.1–2.4 mmol/l) [3, 6]. *n* number of patients in target, *N* number of patients with available values. Cinacalcet mono, subgroup of patients receiving cinacalcet monotherapy at the specific point in time; cinacalcet + high vit D, subgroup of patients receiving cinacalcet plus high dose active vitamin D compounds at the specific point in time; cinacalcet + low vit D, subgroup of patients receiving cinacalcet plus low dose active vitamin D compounds at the specific point in time (low dose active vitamin D was defined as a maximum of 2 µg intravenous paricalcitol three times weekly or equivalent)

The NKF-KDOQI™ as well as KDIGO target achievements were assessed, since the study started only a few months after the publication of the KDIGO guidelines and a certain degree of overlap of adherence to one of these guidelines was expected in clinical practice. Overall, 44.5% of patients (*n* = 134 of 301) reached KDIGO targets and 4.3% (*n* = 13 of 301) reached NKF-KDOQI™ targets for iPTH at baseline, while 65.7% of patients (*n* = 140 of 213) and 30.0% of patients (*n* = 64 of 213) reached the respective targets at month 12. Target achievement for corrected calcium remained stable, with 58.9% (*n* = 142 of 241) and 52.7% (*n* = 127 of 241) at baseline versus 51.9% (*n* = 96 of 185) and 49.7% (*n* = 92 of 185) at month 12, respectively. Phosphorus targets were reached in 18.4% (*n* = 59 of 321) and 45.2% (*n* = 145 of 321) at baseline versus 24.4% (*n* = 57 of 234) and 50.4% (*n* = 118 of 234) at month 12, respectively. Results for the overall group and the subgroups are depicted in Fig. 5 and Figure S1.

### Safety

Safety was not formally evaluated. Within the framework of their pharmacovigilance responsibilities, investigators reported a total of 11 drug-related adverse events in 8 patients (2.4%), 3 of which were considered as serious (dyspepsia, *n* = 2; seroma, *n* = 1).

## Discussion

The TRANSIT study evaluated the different treatment combinations used in clinical practice to treat CKD-MBD. All patients were initiated on a cinacalcet-based regimen. Treatment components, cinacalcet, active vitamin D analogues and phosphate binders, were subsequently adapted to individual patient requirements. We grouped patients according to their individual treatment at each 3‑month interval and described the evolution of treatment patterns and bone mineral markers over time, thus capturing treatment dynamics. An analysis of group dynamics showed that although the overall proportion of patients within each type of treatment remained relatively stable, a substantial proportion of patients switched between treatments. To our knowledge, this is the first report tracing the dynamics of individualized treatment for CKD-MBD in clinical practice over time. On average, approximately 30% of patients changed their regimen from one 3‑month period to any of the other possible treatment types. The reasons for changing a given regimen were to bring to or maintain any of the bone mineral markers within recommended targets and to avoid developments to the extreme (Figure S2).

The present analysis observed daily clinical practice in the participating countries without any study-specific intervention and therefore provides valuable insights into local treatment patterns. Although the trends are similar overall and in the countries, some marked differences were observed. For instance, corrected calcium tended to be higher in Switzerland than Austria, whereas phosphorus tended to be lower (Figure S3). In Switzerland, a substantially larger proportion of patients (37.5%) received cinacalcet monotherapy at baseline, without concomitant active vitamin D analogues, compared to Austria (24.8%; Table 2). In Austria a larger proportion of patients received aluminium-based phosphate binders compared to Switzerland (Table S2). Both NKF-KDOQI™ and KDIGO target achievement rates again were similar in both countries (Figure S4). There are many possible causes for these differences in treatment practice, such as local guidelines and treatment patterns, cost factors and reimbursement rules, differences in diet and phosphorus intake, differences in baseline characteristics or co-morbidities. We have not conducted an analysis of covariates to precisely determine associations between differences in patient-related factors and subsequent evolution of bone mineral marker levels or target achievement. A similarly designed study conducted in several European countries, ECHO, also found marked differences in treatment patterns, biomarker levels and target achievement between countries and provides a discussion of possible reasons [5, 7].

Austria and Switzerland have different reimbursement rules. In the SHPT indications, Austrian health insurances restricted the reimbursement of cinacalcet to dialysis patients with serum PTH above 33.0 pmol/l in whom conventional therapy with phosphate binders and vitamin D analogues the PTH target of 16.5 to 33.0 pmol/l demonstrably could not be reached or maintained. Treatment with cinacalcet may only be extended to a maximum of 6 months in responders with a decrease in serum PTH of >30% after 12 weeks of treatment. Restrictions in phosphate binder type apply. In Switzerland cinacalcet is reimbursed in dialysis patients with SHPT and a PTH above 33.0 pmol/l, when prescribed by a nephrologist. The limitation to second line therapy in Austria may explain the much smaller proportion of patients receiving cinacalcet monotherapy at baseline. Also the differences in the reasons for treatment adaptations (Figures S2) may be justified in the regulatory background.

The baseline characteristics and demographics in the present study were comparable to patients in the Austrian Dialysis and Transplant Registry [6]. Unfortunately, a comparable registry does not exist in Switzerland. According to the Austrian registry, the majority of Austrian centers follow the KDIGO recommendations and they use these recommendations as the basis for their quality assurance initiative. We analyzed both achievement of targets recommended by the NKF-KDOQI™ and KDIGO guidelines. Our analysis showed that more patients (66%) achieved the wider KDIGO target range (12.72 to 63.6 pmol/l) for iPTH compared to the NKF-KDOQI™ recommended range (13.5 to 33.0 pmol/l; 30%). Findings were comparable between Austria and Switzerland by either definition of target. The proportion of patients achieving KDIGO target ranges for iPTH was similar to the findings of the Austrian registry (69%; [6]). The Austrian subanalysis of the ECHO study showed that 36% of patients reached NKF-KDOQI™ targets for iPTH, which is similar to our findings [5]. Across Europe, ECHO found a large variability of 13.5% (UK/Ireland) to 40% of patients (Czech Republic/Slovakia) achieving the NKF-KDOQI™ target for iPTH [7].

Compared to NKF-KDOQI™ (1.13 to 1.78 mmol/l), KDIGO guidelines have a narrower target range for phosphorus (1.13–1.48 mmol/l, defined as the “normal” range of assays commonly used in Austria) and fewer patients (24%) reached phosphorus targets as recommended by KDIGO than compared to NKF-KDOQI™ (50%). Our findings compare positively to the ECHO study, where NKF-KDOQI™ target achievement for phosphorus ranged between 26.2% (CEE country cluster) and 59.4% (Italy). It is a common finding for Austria that phosphorus levels tend to range around the upper limit of the target range. In the registry, 24% of patients are within the phosphorus target range, with 66% being above. In Switzerland, however, phosphorus levels trended to be lower compared to Austria and more Swiss than Austrian patients achieved either target definition.

Approximately 50% of patients remained in the NKF-KDOQI™ range for corrected calcium across the entire study. Since the target range used to represent KDIGO recommendations of the “normal” range (2.1 to 2.4 mmol/l, not rounded, personal communication R. Kramar, president of the Austrian dialysis and transplant registry, Klinikum Wels-Grieskirchen, Austria) is slightly wider on the upper limit than the NKF-KDOQI™ range (2.1–2.37 mmol/l), more patients reached KDIGO targets at baseline (58.9%) compared to NKF-KDOQI™ (52.7%), with a slight decline in target achievement over time (51.9%, KDIGO; 49.7%, NKF-KDOQI™). In the ECHO study, between 40.8% (UK/Ireland) and 59.3% (Nordic country cluster) reached NKF-KDOQI™ targets for corrected calcium. Slightly fewer patients reached calcium targets in Switzerland compared to Austria. Calcium reduction is a known effect of cinacalcet.

This study has some special features. In the attempt to capture the dynamics of SHPT treatment, at each of the evaluated timepoints each patient was newly assigned to the group that best reflected their treatment type. This implies that the welfare of patients receiving a particular treatment type, as a group, is not followed longitudinally. The composition of the groups and thus their patient characteristics is different at each timepoint. This type of analysis provides a very clear picture of everyday clinical practice and suggests that minute adaptations in treatment composition may be partly responsible for treatment success. It does, however, not allow conclusions on the relative benefit of one particular treatment type over the other to be deduced. This question can only be answered in an interventional study where patients are assigned to specific treatment types and maintained over time.

In summary, in Austrian and Swiss clinical practice, cinacalcet is commonly started in combination with active vitamin D analogues. Overall, iPTH trended to increase prior to cinacalcet initiation and decreased afterwards by 43–58% compared to baseline and 66% of patients achieved KDIGO targets for iPTH at study end. Patients’ treatment combinations were constantly adjusted to their needs at each timepoint to optimize individual outcomes. IPTH increased prior to cinacalcet initiation, despite treatment with conventional therapies such as vitamin D analogues and phosphate binders; phosphorus also showed a rising trend. After initiation of cinacalcet iPTH and phosphorus decreased to levels within the recommended target range. Treatment was highly dynamic and tailored to individual needs.

## Conclusion

The results of this study suggest that cinacalcet is a useful treatment component to achieve recommended targets. The details of the treatment mix are subject to individual patient requirements and should be re-assessed on a regular basis.

## Caption Electronic Supplementary Material

Supplementary material including per country analyses, and KDIGO and NKF-KDOQI™ target achievement data
