# Supplementary material for: Management of secondary hyperparathyroidism: practice patterns and outcomes of cinacalcet treatment with or without active vitamin D in Austria and Switzerland – the observational TRANSIT Study
Source: Wien Klin Wochenschr. 2017 Jan 13;129(9):317–28. doi: 10.1007/s00508-016-1153-z (PMC5429358; doi:10.1007/s00508-016-1153-z)
Supplement: Supplementary file 1 — Supplementary material including per country analyses, and KDIGO and NKF-KDOQI™ target achievement data [file 508_2016_1153_MOESM1_ESM.docx]

**Management of Secondary Hyperparathyroidism: Practice Patterns and Outcomes of Cinacalcet Treatment with or without Active Vitamin D in Austria and Switzerland – the Observational TRANSIT Study**

**Supplemental material**

**Table S1. Proportion of patients receiving possible regimen types**

|  | Overall, n (%) | | | Austria, n (%) | | | Switzerland, n (%) | | |
| --- | --- | --- | --- | --- | --- | --- | --- | --- | --- |
|  | Baseline N=333 | Month 6 N=308 | Month 12 N=241 | Baseline N=165 | Month 6 N=156 | Month 12 N=128 | Baseline N=168 | Month 6 N=152 | Month 12 N=113 |
| cinacalcet mono | 104 (31.2)* | 79 (25.6) | 53 (22.0) | 41 (24.8)* | 34 (21.8) | 25 (19.5) | 63 (37.5)* | 45 (29.6) | 28 (24.8) |
| cinacalcet + low dose vitamin D | 95 (28.5) | 73 (23.7) | 56 (23.2) | 43 (26.1) | 36 (23.1) | 32 (25.0) | 52 (31.0) | 37 (24.3) | 24 (21.2) |
| cinacalcet + high dose vitamin D | 126 (37.8) | 113 (36.7) | 83 (34.4) | 79 (47.9) | 63 (40.4) | 45 (35.2) | 47 (28.0) | 50 (32.9) | 38 (33.6) |
| Cinacalcet + other vitamin D | 8 (2.4) | 10 (3.2) | 6 (2.5) | 2 (1.2) | 5 (3.2) | 3 (2.3) | 6 (3.6) | 5 (3.3) | 3 (2.7) |
| vitamin D mono† | - | 22 (7.1) | 26 (10.8) | - | 11 (7.1) | 16 (12.5) | - | 11 (7.2) | 10 (8.8) |
| no SHPT therapy† | - | 11 (3.6) | 17 (7.1) | - | 7 (4.5) | 7 (5.5 | - | 4 (2.6) | 10 (8.8) |

*primary outcome measure (proportion of patients initiating cinacalcet without concomitant active vitamin D analogues); †patients receiving vitamin D monotherapy or no SHPT therapy first appear at month 3

**Table S2. Usage of cinacalcet, vitamin D analogues, and phosphate binders**

|  | **Baseline N (overall) =333** | | **Month 12 N (overall) =241** | |
| --- | --- | --- | --- | --- |
|  | n (%) | Mean weekly dose* | n (%) | Mean weekly dose* |
| **Cinacalcet** | | | | |
| Overall | 333 (100) | 30.7 mg (95% CI ± 0.83) | 198 (82.2) | 45.4 mg (95% CI ± 3.37) |
| Austria | 165 (100) | 32.0 mg | 105 (82.0) | 46.8 mg |
| Switzerland | 168 (100) | 29.4 mg | 93 (82.3) | 43.8 mg |
| **Active vitamin D analogues**** | | | | |
| Overall | 229 (68.8) | 10.8 mcg (95% CI ± 0.96) | 171 (71.0) | 10.7 mcg (95% CI ± 0.97) |
| Austria | 124 (75.2) | 11.1 mcg | 96 (75.0) | 10.2 mcg |
| Switzerland | 105 (62.5) | 10.5 mcg | 75 (66.4) | 11.4 mcg |
| **Phosphate binders, overall** | | | | |
| Overall | 214 (64.3) | - | 156 (64.7) | - |
| Austria | 100 (60.6) | - | 78 (60.9) | - |
| Switzerland | 114 (67.9) | - | 78 (69.0) | - |
| **Calcium-based phosphate binders** | | | | |
| Overall | 159 (47.7) | 2066 mg (95% CI ± 154.1) | 118 (49.0) | 2103 mg (95% CI ± 215.4) |
| Austria | 52 (31.5) | 2296 mg | 52 (40.6) | 2123 mg |
| Switzerland | 107 (63.7) | 1968 mg | 66 (58.4) | 2088 mg |
| **Aluminium-based phosphate binders** | | | | |
| Overall | 52 (15.6) | 2515 mg (95% CI ± 356.3) | 28 (11.6) | 2630 mg (95% CI ± 546.0) |
| Austria | 39 (23.6) | 2969 mg | 22 (17.2) | 3068 mg |
| Switzerland | 13 (7.7) | 1154 mg | 6 (5.3) | 1025 mg |

*in patients with available dose information (the number of patients with available dose information may be different to the number of patients receiving the respective drug, e.g. in patients receiving vitamin D type other, no dose information was available); **doses of active vitamin D analogues were converted to IV paricalcitol equivalents using the following conversion factors: IV calcitriol (x4); oral calcitriol (x8); oral paricalcitol (x2); IV alfacalcidol (x2); oral alfacalcidol (x4); no conversion for “other type of vitamin D”

**Figure S1. Target achievement at baseline and at month 12, overall and by subgroups**


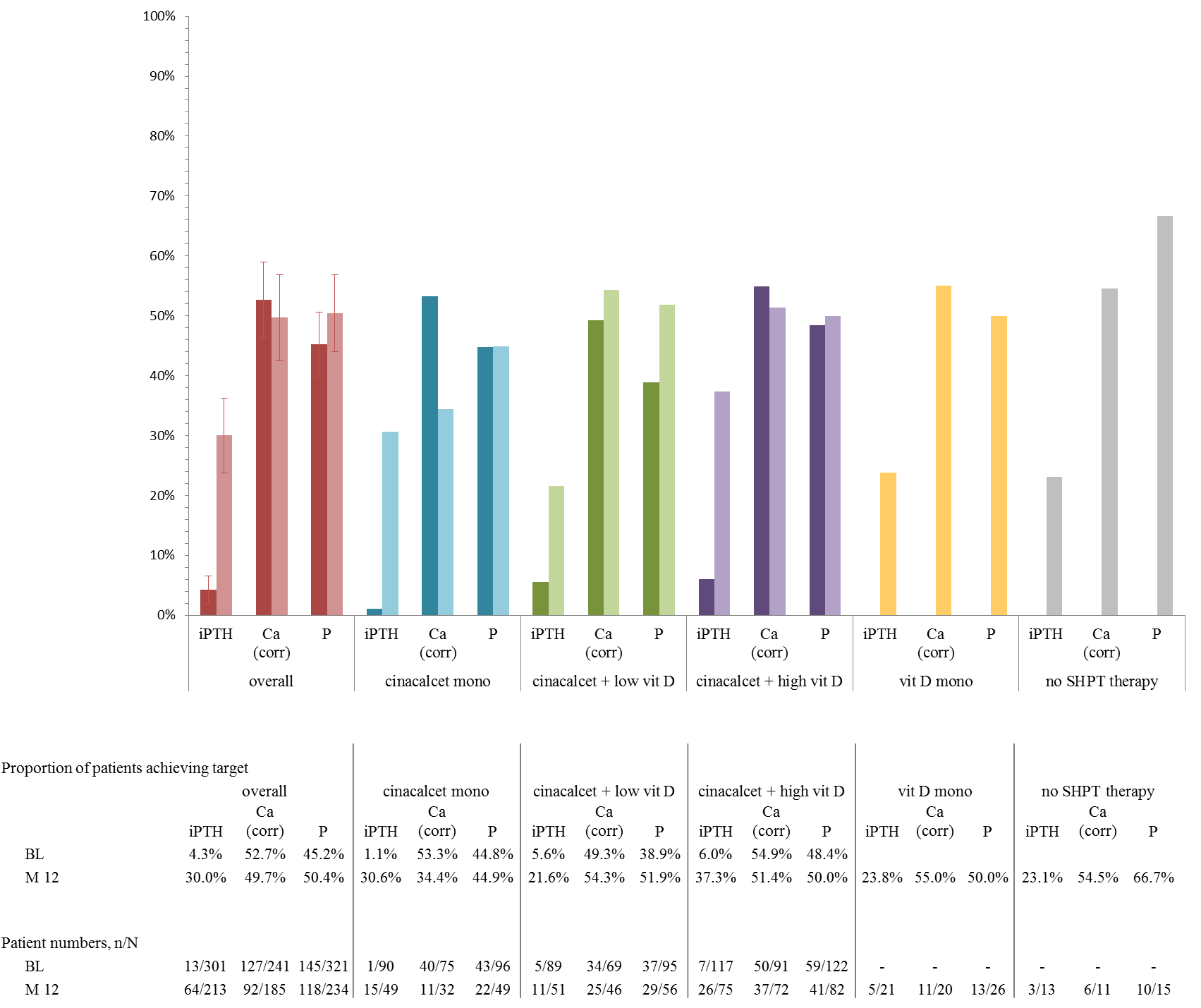


Proportion of patients (95% CI) reaching NKF-KDOQI™ recommended target ranges: iPTH (16.5-33.0 pmol/L), P (1.13-1.78 mmol/L), corrected Ca (2.1-2.37 mmol/L), and corrected Ca x P (<4.44 mmol²/L²)(4)

n, number of patients in target; N, number of patients with available values

**Figure S2. Reasons for treatment adaptations**

A. Primary reason to initiate cinacalcet


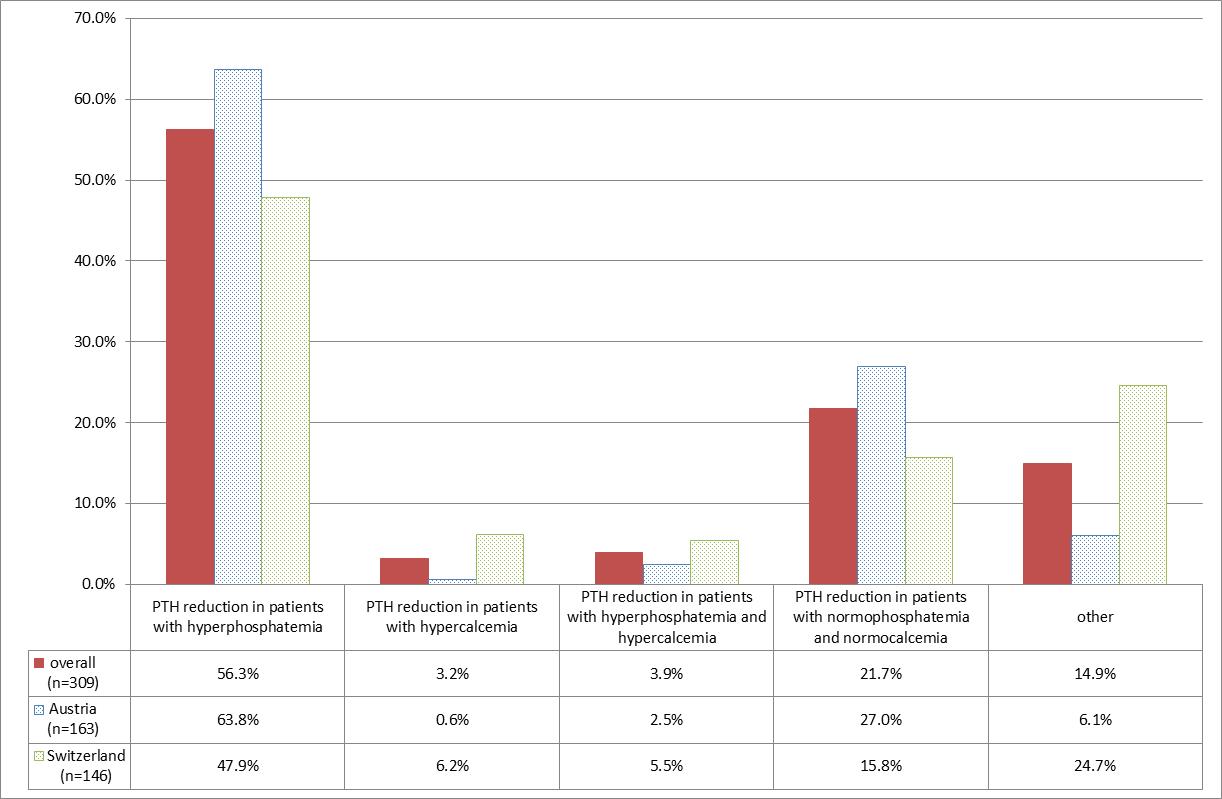


B. Primary reason to discontinue or interrupt cinacalcet


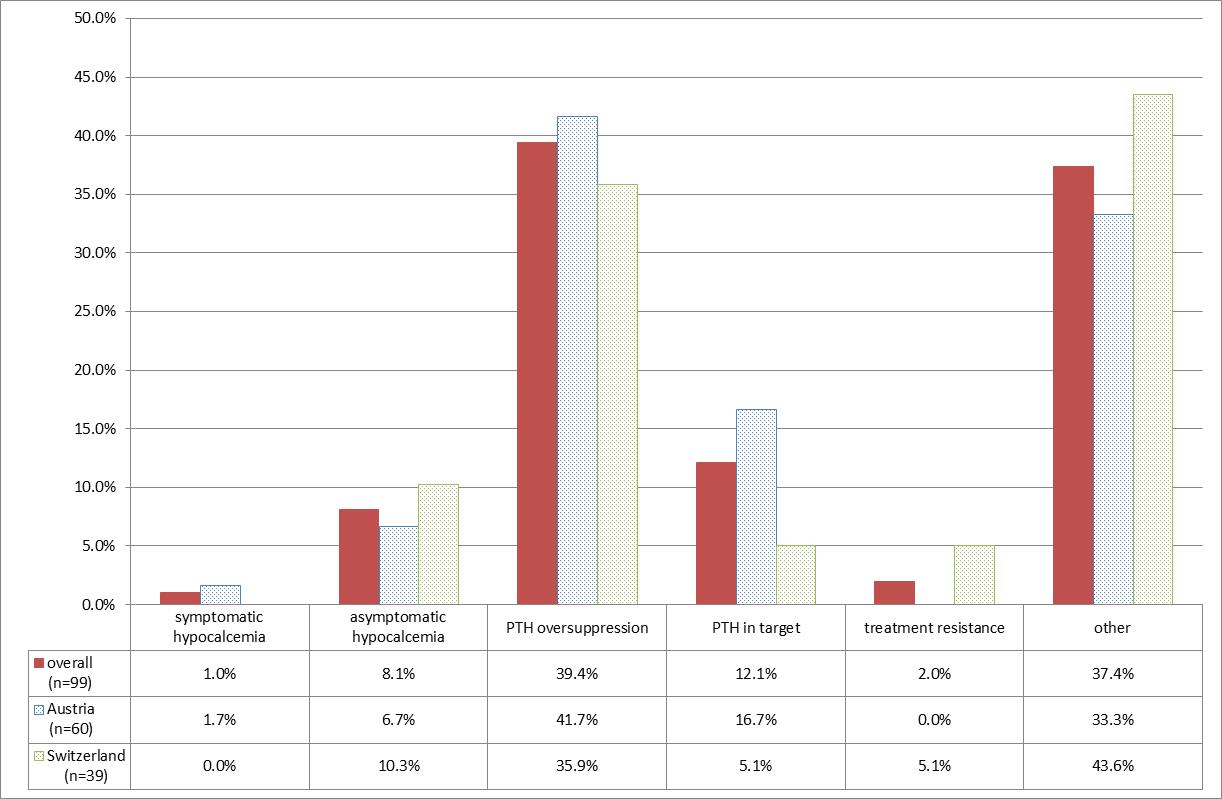


C. Primary reason to re-initiate cinacalcet


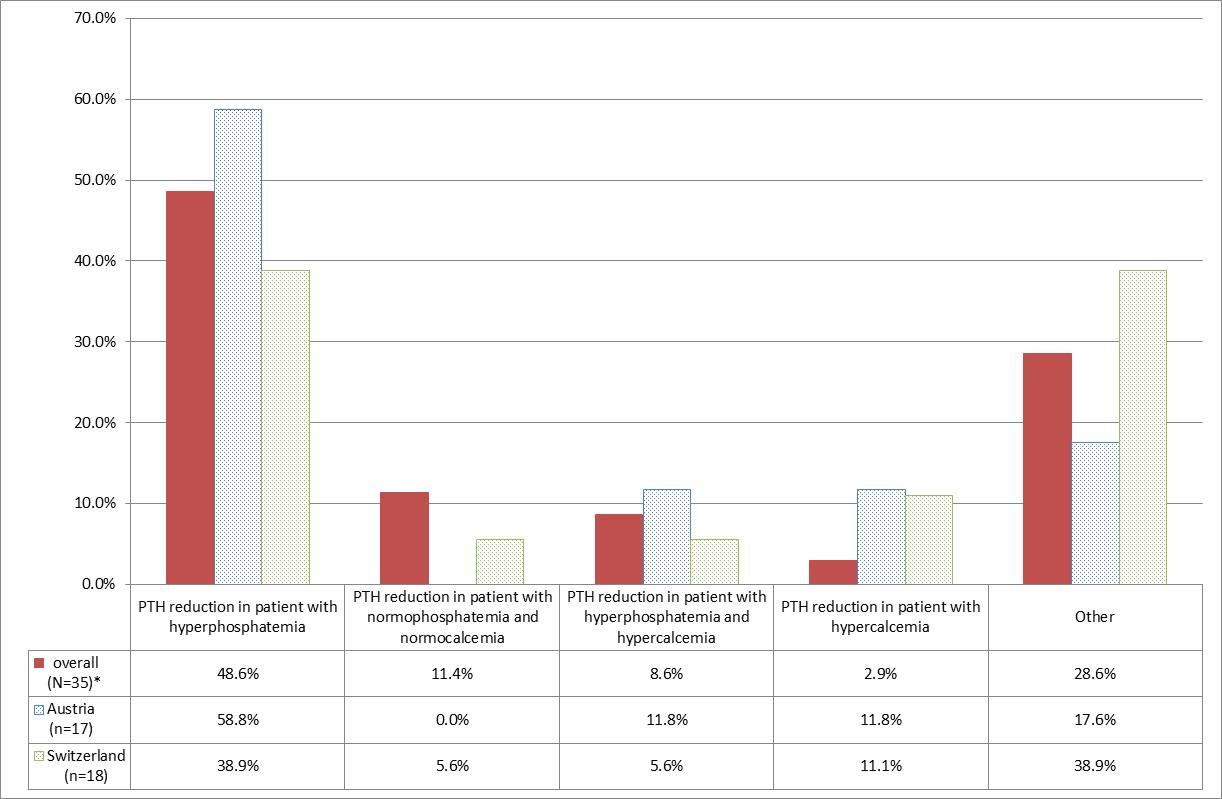


D. Primary reason to initiate active vitamin D analogues


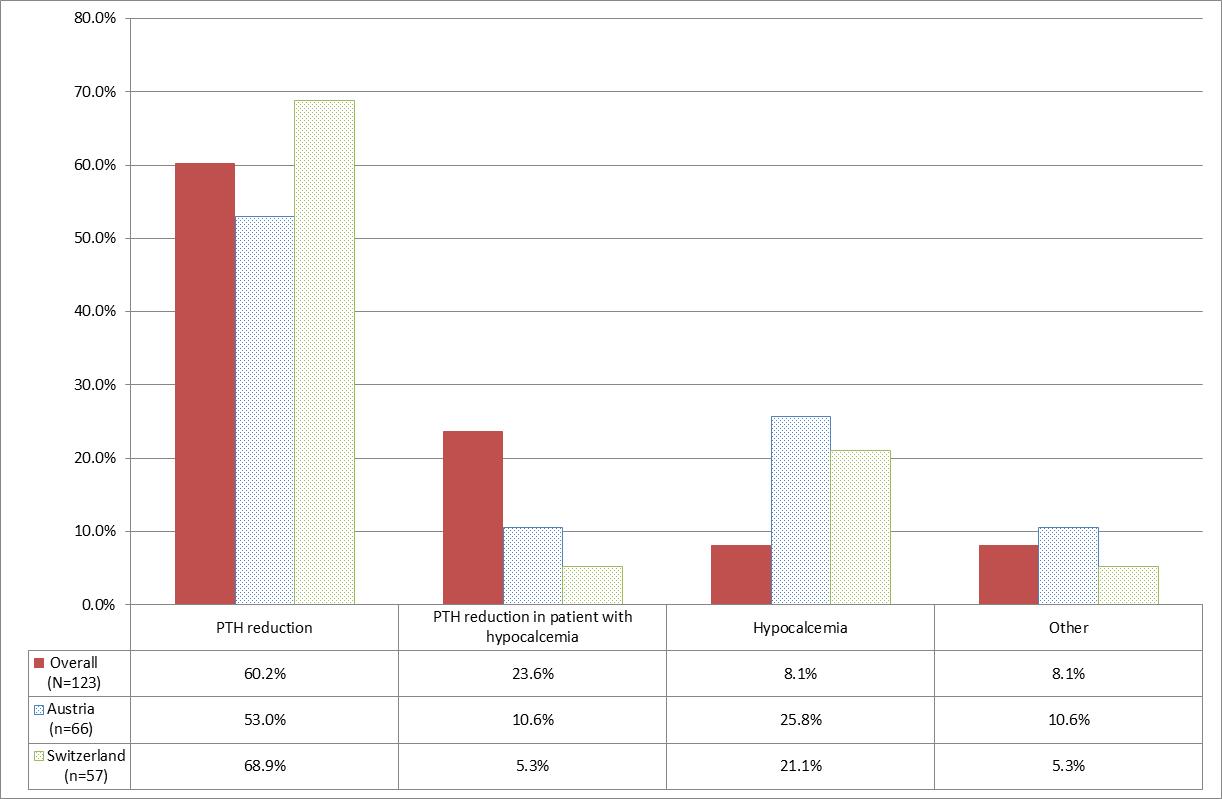


E. Primary reason to discontinue or interrupt active vitamin D analogues


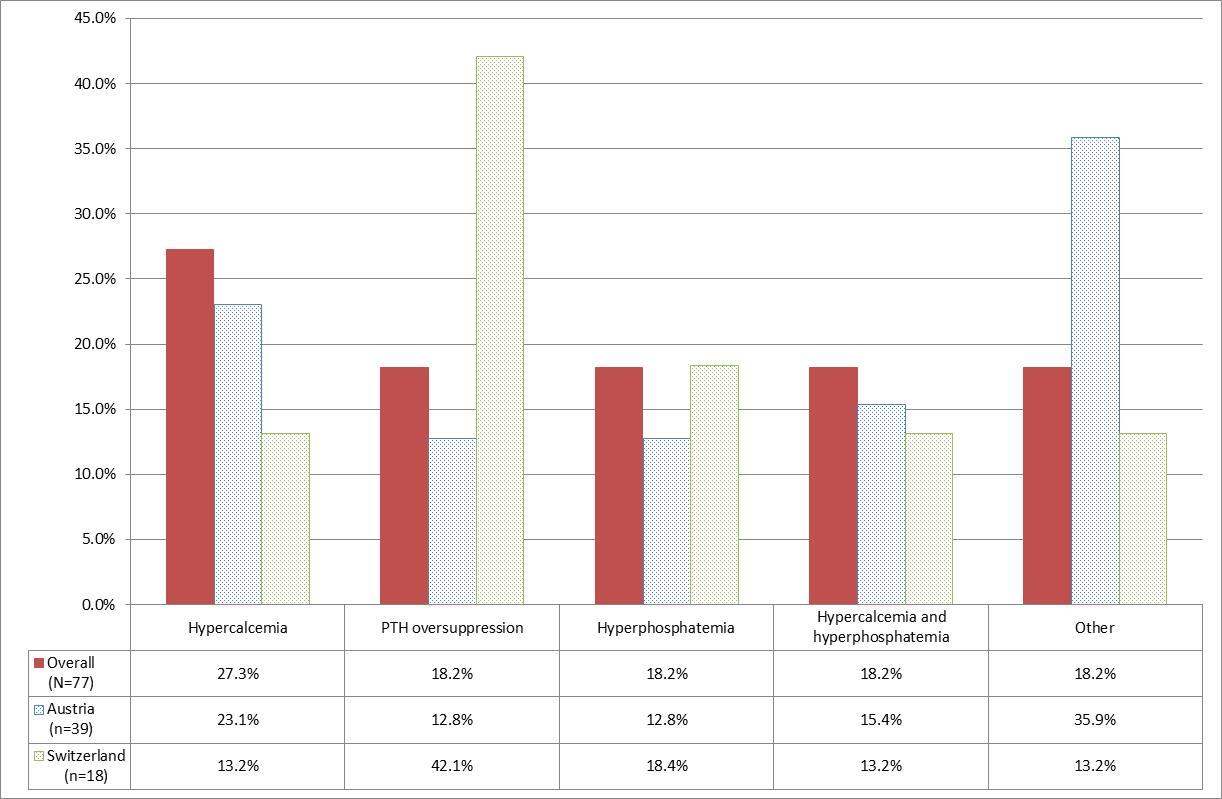


**Country results – Austria and Switzerland**

**Figure S3. Bone mineral markers over time**

A. Mean (95% CI) iPTH over time (pmol/L)


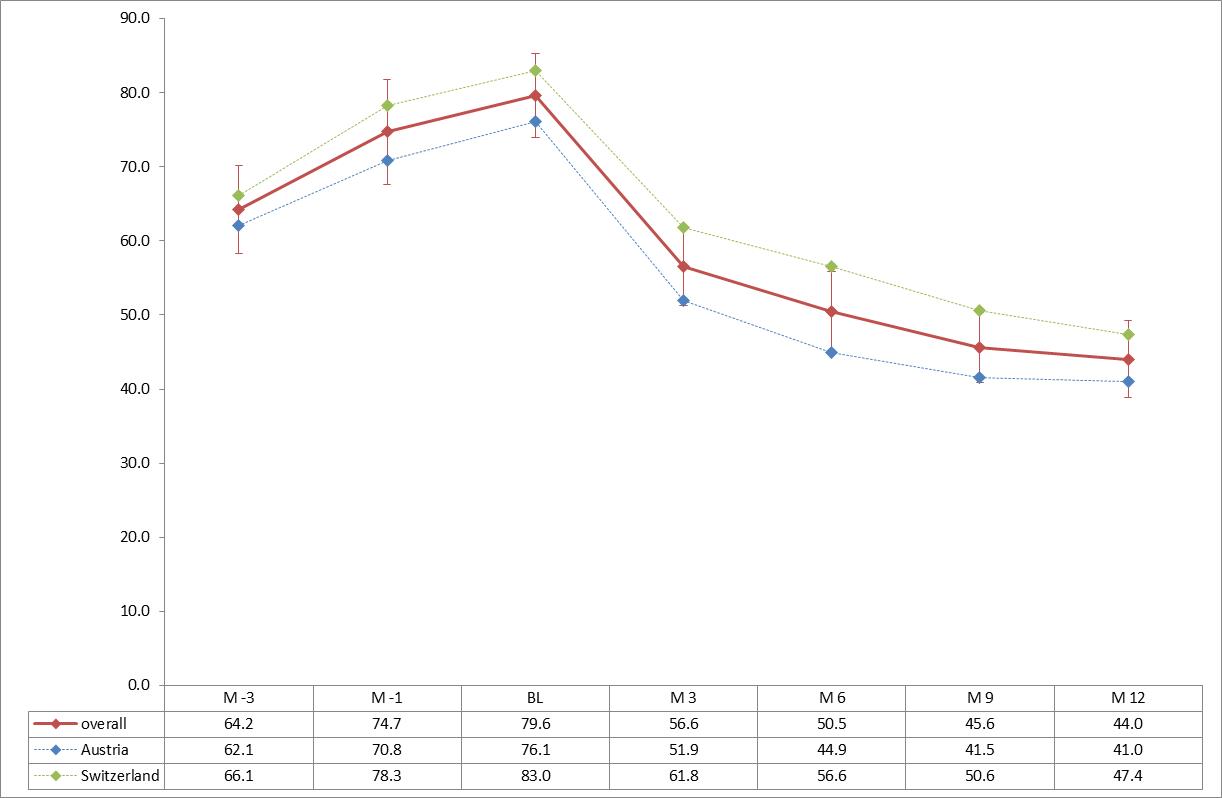


B. Mean (95% CI) calcium (corrected) over time (mmol/L)


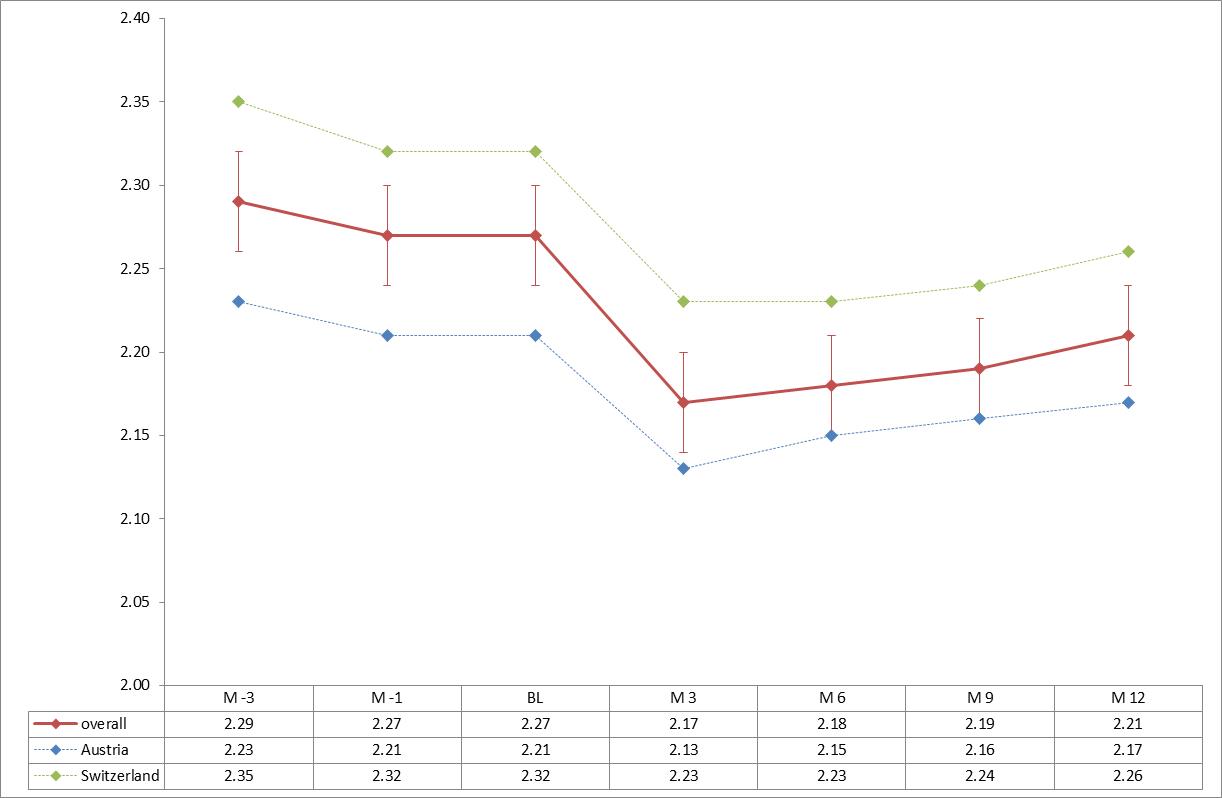


Y-axis does not start at 0

C. Mean (95% CI) phosphorous over time (mmol/L)


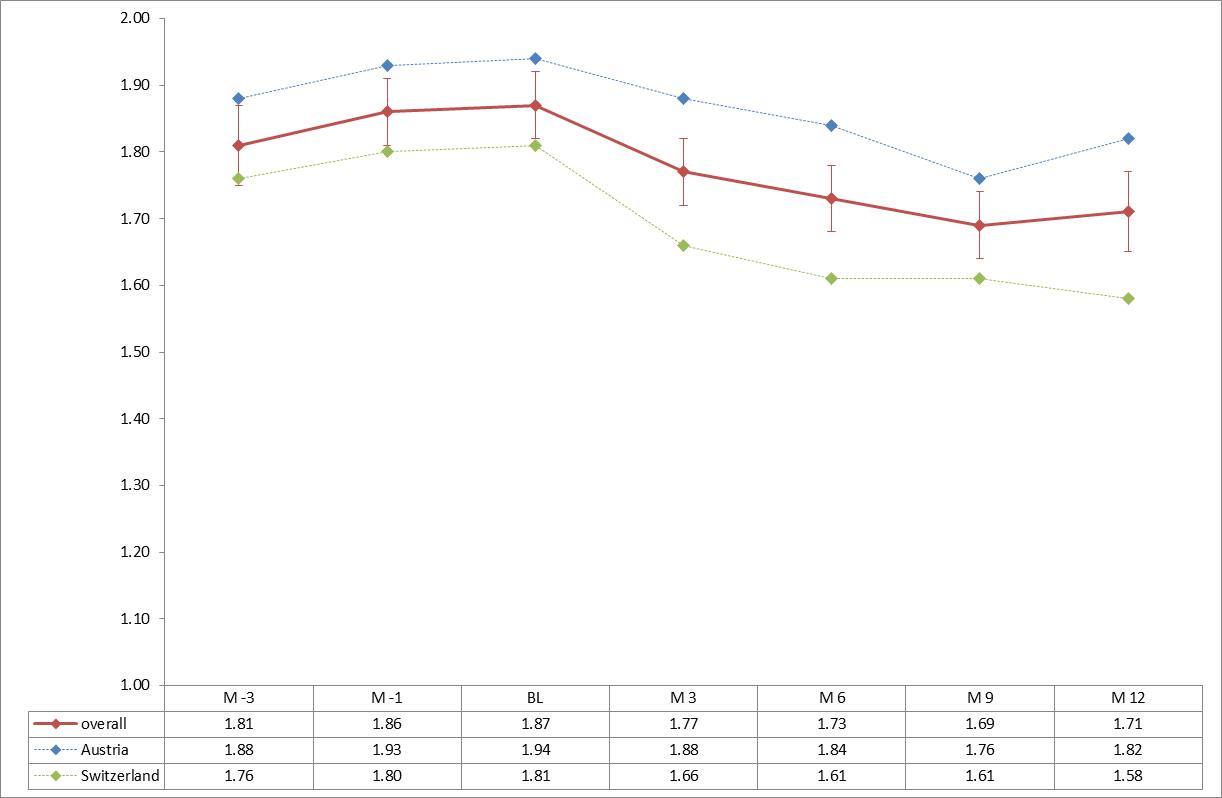


Y-axis does not start at 0

**Figure S4. Target achievement at baseline and at month 12, overall and by subgroups**

A. Proportion of patients (95% CI) reaching NKF-KDOQI™ recommended target ranges*


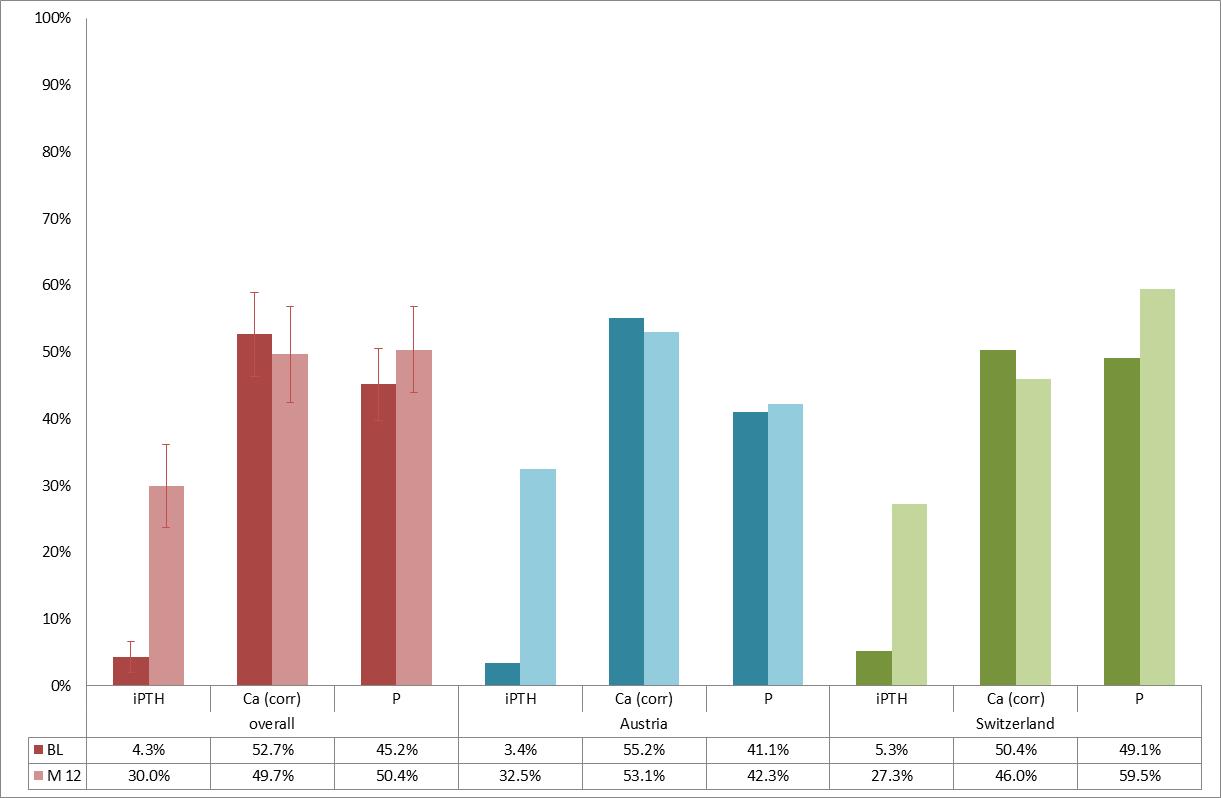


iPTH (16.5-33.0 pmol/L), P (1.13-1.78 mmol/L), corrected Ca (2.1-2.37 mmol/L), and corrected Ca x P (<4.44 mmol²/L²)(1)

B. Proportion of patients (95% CI) reaching KDIGO recommended target ranges*


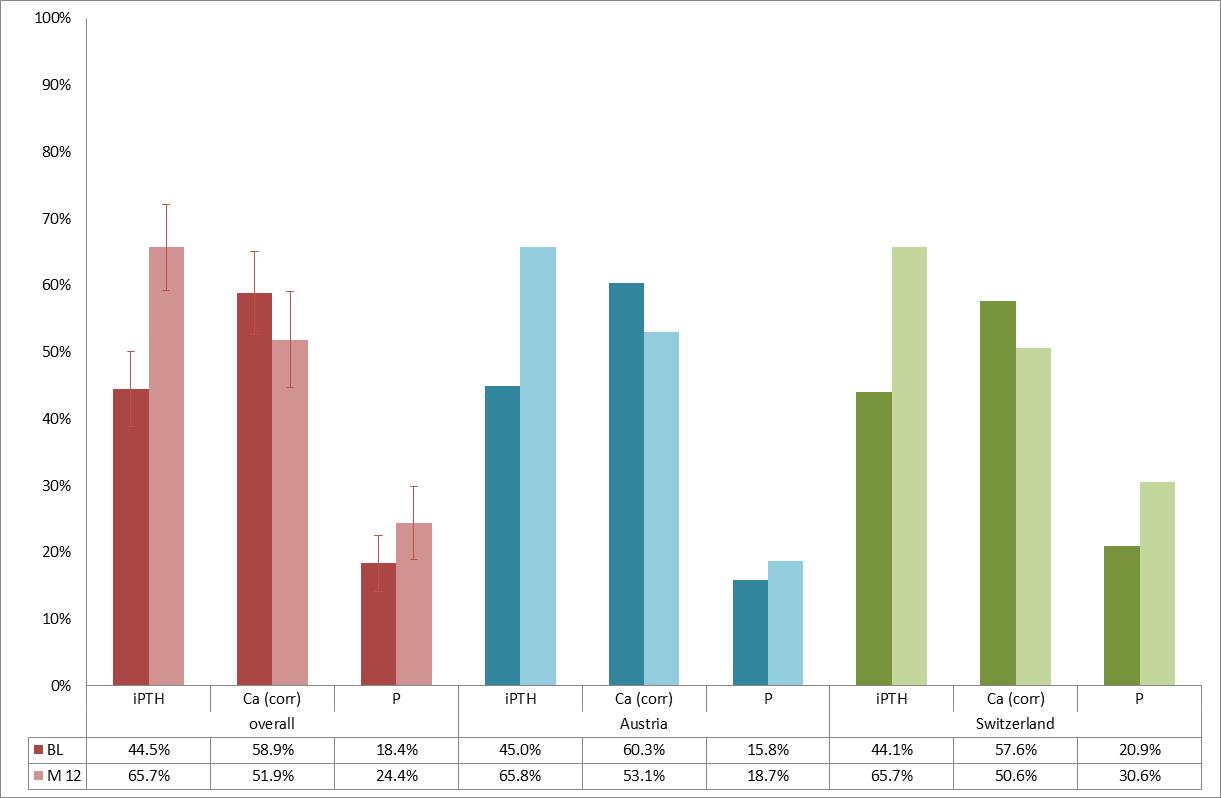


KDIGO recommended target ranges are based on ‘normal’ values from the Austrian dialysis and transplantation registry (5): iPTH (12.72-63.6 pmol/L), phosphorus (1.13-1.48 mmol/L), and calcium (corrected; 2.1-2.4 mmol/L)(2, 3)

References

1. KDOQI. K/DOQI clinical practice guidelines for bone metabolism and disease in chronic kidney disease. Am J Kidney Dis. 2003;42(4 Suppl 3):S1-201.

2. KDIGO. KDIGO clinical practice guideline for the diagnosis, evaluation, prevention, and treatment of Chronic Kidney Disease-Mineral and Bone Disorder (CKD-MBD). Kidney Int Suppl. 2009(113):S1-130.

3. Kramar R. Österreichisches Dialyse- und Transplantationsregister, ÖDTR, Jahresbericht 2012 der Österreichischen Gesellschaft für Nephrologie 2013 [cited 2014 30 April]. Available from: <http://www.nephro.at/oedr2012/oedr2012.htm>.
